# Contact with Mature Cows and Access to Pasture during Early Life Shape Dairy Heifer Behaviour at Integration into the Milking Herd

**DOI:** 10.3390/ani13132049

**Published:** 2023-06-21

**Authors:** Laura A. Field, Lauren M. Hemsworth, Ellen Jongman, Cameron Patrick, Megan Verdon

**Affiliations:** 1 Animal Welfare Science Centre, University of Melbourne, Parkville, VIC 3052, Australia; lauren.hemsworth@unimelb.edu.au (L.M.H.); ejongman@unimelb.edu.au (E.J.); 2 Statistical Consulting Centre, University of Melbourne, Parkville, VIC 3052, Australia; cameron.patrick@unimelb.edu.au; 3 Tasmanian Institute of Agriculture, University of Tasmania, Private Bag 3523, Burnie, TAS 7320, Australia; megan.verdon@utas.edu.au

**Keywords:** behaviour, regrouping, social modelling, social enrichment, pasture-rearing, nanny cows, agonistic, welfare, integration

## Abstract

**Simple Summary:**

Cow–calf separation immediately after birth is an issue of growing concern to global consumers of dairy products. The practice is also increasingly acknowledged by dairy scientists as coming at the detriment of the long-term behavioural, emotional and social development of replacement heifers. When dairy heifers first calve, they must successfully mix for the first time with herds of larger, more dominant, older cows, who can be aggressive and cause stress. Our study used contact with non-maternal adults and access to pasture rather than space-restricted sheds as a means to increase the complexity of the early-life environment of pre-weaned, artificially-reared dairy calves. When heifers reared under these differing conditions were then mixed with mature cows from a commercial milking herd at 23 months of age, the agonistic behaviour of those reared at pasture with adult contact suggested that these heifers were the most dominant within the wider heifer groups, and their feeding behaviour was the most similar to the cow groups into which they were mixed. Interestingly, heifers reared at pasture without adult contact seemed better able to adapt to grazing in a group of mature cows than heifers reared in sheds without adult contact. We suggest that exploring ways to increase both the physical and social complexity of the rearing environment may improve the ability of heifers to successfully integrate into a herd of older, more dominant animals.

**Abstract:**

This study aimed to determine the effects of early-life physical and social enrichment on the ability of dairy heifers to integrate into a herd of mature cows. Fifty heifer calves were reared from the ages of 2–13 weeks in one of three treatments: (1) Hand-reared and group-housed in sheds (CC); (2) Hand-reared and group-housed at pasture (−S); or (3) Hand-reared and group-housed at pasture, with 3 non-familial dry cows per group (+S). At 23 months of age, these heifers were introduced in groups to small herds of cows (Cows) at pasture. Social interactions were recorded continuously for two 1-h periods. Feeding, ruminating and resting behaviours of all animals and walking, standing and lying behaviours of 36 heifers only (+S = 14, −S = 13, CC = 9) were recorded for 48 h after mixing. Heifers that were managed as calves according to the CC treatment delivered less agonistic behaviour to other heifers after mixing than those reared in the +S or −S treatments (*p* = 0.002 and *p* = 0.041, respectively). On Day 2, +S heifers and cows spent the lowest proportion of time feeding (*p* = 0.961), with −S heifers spending significantly more time feeding than cows (*p* = 0.046), while CC heifers spent more time feeding than both +S heifers and cows (*p* = 0.027 and *p* < 0.002, respectively). Increasing the complexity of the early-life environment, particularly socially, may aid heifers in integrating into groups of multiparous cows later in life and shape their lifelong social experiences with same-age conspecifics.

## 1. Introduction

In dairy systems worldwide, common practice has evolved to remove calves from their dams within 24 h of birth and to house adult and juvenile animals separately. Calves are usually reared artificially, and in pasture-based systems, they are typically managed as a single same-age cohort for their first 2 years of life [1]. Juvenile and pre-parturient replacement heifers in pasture-based systems tend to graze dry-land pasture with little intensity [1]. Once they enter the milking herd following calving, they are generally introduced to mature cows for the first time, and the mixed-age milking herd is managed within intensive and competitive grazing regimes [1]. The cow–calf separation system was developed to improve reproductive efficiency and milking ease of the dam, simplify management of both cow and calf and due to the belief that health risks to both cow and calf are more easily mitigated by separation [2]. Maternal contact during early life has, however, been shown to improve social competence across many species, including chickens [3], fish [4], quail [5], rats [6] and rhesus macaques [7]. The effect has also been extensively shown in dairy cattle (e.g., [8,9,10,11,12]). Improving the social complexity of the rearing environment, for instance, by rearing calves in pairs or groups instead of individually, appears to have similar effects [13,14].

Regrouping is a stressful but necessary management practice experienced by most domesticated dairy cattle [15]. For dairy heifers, the most intensive regrouping experience often occurs around the time of primiparturition, when they are first mixed into a larger herd of older, more experienced and unfamiliar animals. Regrouping has previously been associated with decreased feed intake and milk yield and increased social stress and aggression, mainly during the first 1–2 h but up to 15 days after regrouping [1,16]. Agonistic behaviour is particularly directed by older cows towards naïve heifers and other less dominant herd members [15,17,18]. When inhibited from normal social development, animals may fail to learn important social cues, making integrating into a new herd, grazing competitively and forming relationships with unfamiliar animals more difficult [14,19]. 

Successful integration could be indicated by measures such as the synchrony of the group (i.e., the proportion of animals in the group engaged in the same behaviour at the same time) when performing highly motivated behaviours such as feeding and lying, the latency of individuals to feed or lie after regrouping, or the frequency and nature of agonistic interactions experienced by the individual [20,21,22,23,24]. Continuous monitoring systems, such as wearable technologies, permit the collection of whole-day adaptation and synchrony data, while targeted real-time behavioural observations provide insight into the ways that individuals approach social challenges and their competency in dealing with conflict.

Current research, primarily undertaken in indoor housing rather than pasture-based systems, has compared the effects of social enrichment in the early lives of calves through social isolation and pair or group housing or via contact with the dam and other adult herd members, with all forms of social enrichment resulting in improved social competence (e.g., [11,13,14]). Both pair, rather than individual housing, and pre-pubertal adult contact improve social competence and reduce social stress and agonistic interactions in a range of species, including cattle and horses ([25], review: [16]). Less is known about the importance of non-maternal adult cattle for calf development.

This study aimed to determine whether enriching the early-life physical and social environment of dairy heifers with pasture access and non-maternal adult contact would improve social behaviours and ease their integration into the milking herd by providing them with the opportunity to develop and learn species-appropriate behaviour from experienced adults and through more complex physical experiences in early life. We hypothesised that at 23 months of age, heifers reared with non-maternal adults would integrate more easily into a herd of mature milking cows than heifers reared without adult contact. We further hypothesised that heifers reared in more complex physical conditions would integrate more easily than those reared in sheds, but not more than those reared outdoors with adult contact. We believed this would be indicated by a reduced latency to lie and feed, an increased likelihood of feeding synchrony with mature cows, and a reduction in agonistic interactions (delivered and received) with adult cattle.

## 2. Materials and Methods

This experiment was conducted at the Tasmanian Dairy Research Facility (TDRF) near Elliott in north-west Tasmania, Australia (41°08′ S, 145°77′ E; 155m above mean sea level). All animal procedures were approved by the University of Tasmania Animal Ethics Committee (A0018141) under the Tasmanian Animal Welfare Act (1993). 

One of three management protocols was imposed on 60 mixed-breed dairy heifers from the ages of 2–13 weeks between August 2019 and November 2019. These were considered experimental treatments, which were: (1) Hand-reared calves group-housed in sheds (2 commercial control groups of 10 calves, called CC); (2) Hand-reared calves group-housed on 0.5 ha cultivated pasture (2 experimental control groups of 10 calves each, called −S); and (3) Hand-reared calves group-housed on 0.5 ha cultivated pasture and with 3 non-familial dry cows per group (2 experimental treatment groups of 10 calves each, called +S). Ten heifers failed to conceive and were removed from the experimental herd by January 2021 (+S = 4, −S = 3, CC = 3), and wearable technology was fitted to the remaining 50 heifers in May 2021 (technologies described in Section 3.3). 

Integration testing on the 50 experimental heifers used 100 dry cows from the TDRF milking herd. Testing was conducted over two weekends in June and July 2021. The TDRF herd is comprised of several breeds and their crosses, and all animals in this experiment were Friesian, Jersey, Swedish Red, Australian Red or their associated crosses. The breed distribution of the 50 experimental heifers remaining in the replacement heifer herd after 18 months of age reflects this mix (Friesian +S = 10, −S = 9, CC = 6; Jersey +S = 1, −S = 2, CC = 1; FJ, FFFJ, FJJJ or majority FJ x other dairy genetics +S = 5, −S = 6, CC = 10). 

### 2.1. Pre-Testing Period

A timeline detailing the lifetime management of each treatment is outlined in Table 1. Full details of +S and −S rearing conditions are described in Field et al., 2023 [26].

The original 60 heifer calves were born within 35 days of each other and identically managed for their first two weeks of life. All calves were born at pasture and separated from their dams within 12 h of birth. They were fed 2 L of quality colostrum twice within 24 h of birth (total 4 L) after being relocated from the calving paddock to woodchip-bedded group housing pens containing 12 calves each, after which they were fed 2.5 L of whole milk twice a day from 12-teat feeders. Calves in all experimental treatments had *ad libitum* access to water and calf starter pellets from birth and for the duration of the experimental period.

When calves were 14–18 days of age ($\bar{x}$ = 16.35 days), 40 were allocated to one of the two +S or −S groups, balanced for age, breed, and weight. Treatments were applied over two time-replicates commencing exactly 1 week apart (*n* = 20 calves per treatment/replicate). The non-familial dry cows housed with +S groups were empty, dry, mixed-breed multiparous cows from the TDRF milking herd. Each group was housed on approximately 0.5 ha of cultivated ryegrass pasture and offered 3 L whole milk twice daily on 10-teat milk feeders. All paddocks contained a water trough and a creep area approximately one-quarter the length of the paddock and accessible only by calves, containing a three-sided shelter measuring approximately 2.5 m × 2 m (see [26] for a visual depiction of the experimental paddocks). Barriers were erected such that calves had visual contact only with animals in their own group. All +S and −S calves were reared in these assigned groups outdoors in paddocks until the youngest calf in each replicate (time-based) was 12 weeks of age. Cows were then removed from +S paddocks, out of visual and auditory contact, and calves were gradually weaned over the following week. Calves remained in their treatment groups at pasture with continued access to water and calf starter concentrate throughout the weaning process. At the completion of weaning, the youngest calf was 13 weeks old.

The 20 CC calves were managed commercially by the farm for the duration of their rearing period. Breed distribution was similar to that of the +S and −S groups, with a slightly higher number of crossbred animals. They were housed in wood-chip-bedded pens in groups of 10–12, and from the age of 2 weeks, they were fed 5 L whole milk once daily on 12-teat milk feeders. +S and −S calves were offered 1 L more milk than their CC counterparts to ensure sufficient nutritional support for outdoor housing during the winter and early spring. CC calves were gradually weaned at 12 weeks of age.

Following weaning at approximately 13 weeks of age, all heifers were mixed at pasture and continued to be housed outdoors and rotationally grazed on rainfed pasture. In October 2020, at approximately 13 months of age, all heifers on the TDRF farm were submitted to the farm breeding program. By 18 months of age, 10 of the original 60 heifers had been removed from the experiment for reproductive failure or not meeting the farm’s genetic requirements, leaving 50 heifers remaining on the farm for testing. 

### 2.2. Fitting of Precision Behavioural Monitoring Technology

In May 2021, each of the retained 50 experimental heifers was restrained in a crush and fitted with a MooMonitor+ collar (Dairymaster, Tralee, Ireland). MooMonitor+ collars have been validated to measure grazing behaviour in pasture-housed dairy cows and also collect data on activity, resting, and rumination in 15-min increments [27]. Resting, as defined by the MooMonitor+ collars, does not preclude resting while standing and is therefore not synonymous with lying behaviour.

Thirty-six heifers (+S = 14, −S = 13, CC = 9) were also fitted with a RumiWatch pedometer (RWS; Itin and Hoch GmbH, Liestal, Switzerland) on their right hind leg above the metatarsophalangeal joint as instructed by the manufacturer. RumiWatch pedometers use a three-dimensional accelerometer and have been validated to continually record the frequency and duration of time spent lying, standing, and walking [28]. Heifers acclimatised to these devices over the following weeks. Collar and pedometer straps were regularly monitored for rubbing and discomfort.

### 2.3. Integration Testing: Experimental Design 

At 23 months old, the 50 experimental dairy heifers plus the additional 100 multiparous dairy cows participated in integration testing. All animals on the farm were dry during testing and were due to calve between July and October 2021. Approximate calving dates were calculated for all cows and heifers in January 2021 by a veterinarian conducting pregnancy diagnosis through transrectal palpation. 

The 50 heifers were divided into two time-replicates for testing, according to their expected calving date (i.e., within the first (early calving, EC) or second (late calving, LC) calving peak, estimated to commence approximately 2 weeks apart). The EC peak comprised 23 heifers, with 9 CC heifers, 8 −S heifers and 6 +S heifers. The LC peak comprised 27 heifers, with 8 CC heifers, 9 −S heifers and 10 +S heifers. Replicates underwent integration testing approximately 1 month prior to their earliest estimated calving date. The two replicates were therefore tested two weeks apart. Prior to integration testing, all heifers were managed as a single herd, regardless of their estimated calving date. 

Cows were expected to commence calving approximately the same week as LC heifers, with a second extended calving peak expected approximately 3 weeks after this. Cows ranged in age from 3 to 9 years, having completed between 1 and 7 lactations. At the time of testing, cows were managed in two groups: early calvers and late calvers, to account for their different nutritional needs. Cows were selected for integration testing according to these two groups, wherein early-calving cows were paired with EC heifers and late-calving cows were paired with LC heifers. All cows on the TDRF farm are fitted with MooMonitor+ collars for their productive lives, and thus data from these devices was also collected for the mature cows for the 48 h of integration testing.

The testing protocol was identical for each time-replicate (outlined in Supplementary Table S1). Within each replicate, heifers were allocated to one of three groups (total = 6 groups), ensuring groups were balanced for heifer treatment (i.e., +S, −S or CC) and rearing group (i.e., groups 1–6 during rearing). The resulting heifer group size ranged from 6 to 11 heifers. Heifers due to be tested were drafted from the larger replacement heifer herd 3 days prior to testing. Each heifer was sprayed with an identifying colour and number and drafted into their individual groups. For the next three days, heifers acclimatised to their smaller groups at pasture, out of view of the testing paddocks.

**Figure 1:**
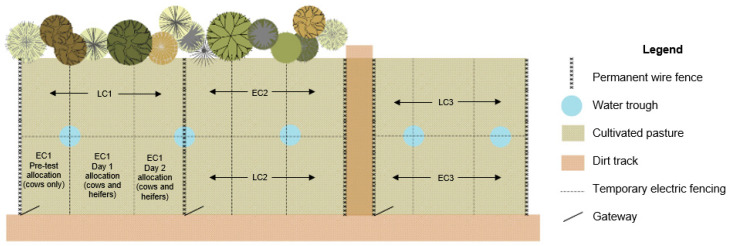
Paddock allocation for experimental groups (not to scale). EC = early calving time replicate; LC = late calving time replicate. Each replicate comprised 3 groups, as illustrated in this figure. The EC1 paddock illustrates in greater detail the three allocations (one for cows only on the day prior to testing and one each for the two testing days for the mixed group of heifers and cows).

Each group was assigned to a different paddock for testing (see Figure 1). The three adjacent paddocks contained cultivated perennial ryegrass. One half of each paddock was used in each replicate. The paddocks were split into 3 separate allocations of pasture using electrical tape, with one allocation of pasture offered on each of the three days of testing. Pasture allocations were calculated at 75 m^2^ of fresh pasture per animal per day, ensuring that although group sizes varied, stocking density remained stable. Pasture coverage was estimated using a plate metre the day prior to testing, and across all paddocks for each replicate, there was an estimated average of 2800 kg DM/ha. Figure 1 illustrates the paddock layout, including the division of paddocks as described above. Pasture allocations were back-fenced when animals were offered fresh allocations, so animals could not access residual pasture from the previous day. Fresh water was available at all times. Weather was similar across all testing days (maximum temperature range 12.7–13.3 °C, minimum temperature range −0.6–4.2 °C, daily rainfall range 0–11.8 mm, with all days of testing experiencing some light wind and cloud, as per the Australian Bureau of Meteorology).

On the day before each weekend of integration testing, cows were randomly allocated to the 3 heifer groups at a ratio of 2 cows to every heifer, balancing for cow age and lactation number. The resulting groups of cows ranged from 12 to 22 animals. Table 2 outlines the composition of each group. Cows were marked with stock paint across the hips and withers before being moved to their allocated paddocks (pre-test allocation, as per Figure 1) and given 24 h to acclimatise. During this period, heifers remained in their allocated groups, out of view. 

### 2.4. Integration Testing: Behavioural Observation Data Collection

The protocol for behavioural observations during integration testing, including a detailed ethogram for expected social behaviours (Table 3), was developed in reference to the protocol outlined in Boyle et al. [21]. The ethogram was developed so that all behaviours (except for mutual allogrooming or head-to-head contact) could be easily identified as being actively delivered or received. Displacements were defined as contact-free interactions in which the receiving animal moved away from the delivering animal. 

Integration testing was conducted over two days. Direct observations were made on each paddock consecutively for one hour on both days of testing. On each day, each heifer in the paddock was randomly allocated a single observer, who observed this heifer, identifiable by the number sprayed in coloured stock spray on her side, continuously. All the heifers in the paddock were thus observed at once. Observers stood around the perimeter of the daily pasture allocation. Groups were observed in order, 1–3, on each testing day. Observers had been trained together with the experimental ethogram and videos of social interactions of cattle grazing at pasture; an inter-observer reliability analysis using the Kappa statistic was performed to determine consistency among observers. Inter-observer reliability was calculated as Kappa = 0.761 (*p* < 0.001), 95% CI (0.574, 0.948). 

On day 1, resident cows were moved into the fresh allocation of grass. The corresponding group of heifers was then introduced into the pasture allocation already inhabited by the older resident cows. Observations began when the final heifer entered the allocation of grass. For each heifer, data was collected for every behaviour exhibited by the heifer as listed in the ethogram presented in Table 3, at a count level in 6 × 10-min blocks. For each behaviour delivered or received by the heifer, the group (i.e., heifer or cow) of the partner animal was recorded. This resulted in a total of an hour of continuous observation post-mixing, allowing the frequency with which each heifer delivered or received agonistic and affiliative behaviours from/to cows and from/to other heifers to be calculated. Observers were blind to the heifer treatment. This protocol was repeated for the following two paddocks in each replicate. On Day 2, the now mixed group of cows and heifers was moved to the fresh allocation of pasture and observed for 1 h following the same protocol. 

### 2.5. Integration Testing: Precision Behavioural Monitoring Technology Data Collection

RumiWatch pedometers collected data on Lying, Standing and Walking behaviours as a total proportion of time per 10 min. Latency to lie was calculated as the duration of time from the beginning of data collection for the day (heifers entering the paddock on Day 1; the group was moved to fresh grass allocation on Day 2) to when the RumiWatch pedometer of each animal first recorded lying behaviour.

MooMonitor+ collars, fitted to all 50 heifers and 100 cows, continuously collected data on Ruminating, Resting, Feeding and ‘Other’ behaviours as a total proportion of 15-min blocks. MooMonitor+ data were missing for all +S heifers and 50% of all other animals from Paddock 3 in replicate 2 due to a technical malfunction associated with the topography of the testing site. Therefore, all MooMonitor data from this paddock was removed from the analysis. (+S *n* = 4, −S *n* = 4, CC *n* = 3, Cow *n* = 11). Data from a further 2 cows and 1 CC heifer in time-replicate 1 and 2 cows in time-replicate 2 were missing and not included in the analysis.

### 2.6. Statistical Analysis

Day 1 (post-mixing) and Day 2 (post-fresh pasture allocation) data were analysed separately for all measures. Data from pedometers and MooMonitor+ collars was collected from when heifers first entered the testing paddock (Day 1) or were moved onto fresh pasture (Day 2). Twenty-four hours of data were analysed for Day 1 and 12 h for Day 2. Heifers began lead-feed in preparation for calving on the day immediately following testing; this timeframe, therefore, accounted for heifers being removed from testing paddocks early in the morning of the day after Day 2 testing. Day 1 and Day 2 data did not overlap at any time. 

In the following descriptions of the statistical analysis, ‘Heifer’ denotes the individual animal from whom data was collected. ‘Treatment’ denotes +S, −S, CC, and in the case of MooMonitor+ data, also cows. ‘Time replicate’ denotes whether heifers were tested as part of the EC or LC group, and ‘Day’ accounts for testing Day 1 or 2, and ‘Paddock’ describes the specific testing group the heifer was assigned (i.e., 1–6). ‘Group’ was included in all statistical models to account for the individual rearing group a heifer belonged to (1–6). Significant differences were determined at *p* < 0.05.

#### 2.6.1. Statistical Analysis: Behavioural Observations

Data were analysed at the heifer level, and all behaviours were analysed in one model. A generalised linear mixed model was used to model the effect of treatment on the frequency of each behaviour per observation day. The glmmTMB package version 1.1.4 [29] (Brooks et al., 2017) in R version 4.2.1 (R Foundation for Statistical Computing, Vienna, Austria) was used.

Allogrooming and Cohesive behaviour on Days 1 and 2 were removed from analysis, along with Investigative behaviour on Day 2, due to almost all of the observed counts being zero. Butting and Displacement behaviours were aggregated to form an umbrella ‘Agonistic’ behaviour for analysis. This was further aggregated with the variable ‘Mutual head-to-head contact’ to create the measure of ‘Total Agonistic Behaviour’, which was also analysed. 

The model included the random effect of heifers within a paddock within a time-replicate, which accounted for repeated measures on these heifers across days. An additional random effect of grouping was also included. This random effect structure is represented in R syntax as “behaviour:time_rep/paddock/heifer” and “behaviour:group”. There were issues with convergence when separate GLMM models were used for each behaviour, particularly for those with low frequencies. To stabilise the analysis, all behaviours were analysed in a single GLMM that assumed the same shape parameter (variance) for all behaviours and days. Tests for treatment differences within each day and behaviour were performed. Fixed effects of behaviour, observation day, and treatment were included in the model, along with all interaction terms. The count (frequency) of the observations was the outcome of the model. Since the outcome was a whole-number count, a negative binomial response distribution with a log link was used. This distribution allows for overdispersion and is adequately modelled in the present dataset. Where significant treatment effects were determined by the model, pairwise comparisons are reported to further describe these effects. Data describing means, overall tests and pairwise comparisons for behaviour measures are presented in Supplementary Tables S2–S4.

#### 2.6.2. Statistical Analysis: Technology

For both RumiWatch and MooMonitor+ data, linear mixed-effects models were fitted per testing day to the proportion of time spent in each behaviour, and the latency to lie separately. The lmer package version 1.1 [30] in R version 4.2.1 (R Foundation for Statistical Computing, Vienna, Austria) was used to fit all models. Treatment, day, and time-replicate were included as fixed effects, along with an interaction between treatment and day. Group, paddock and heifer were included as random effects, with heifer or cow nested inside paddock.

For MooMonitor+ data, the estimated variance component of the group random effect was zero, resulting in model convergence warnings, so the group effect was removed from the final models. Where significant treatment effects were determined by the model, pairwise comparisons are reported to describe these effects. Data describing means and pairwise comparisons for worn technology measures, including latency to lie, are presented in Supplementary Tables S5–S11 and Supplementary Figure S1.

#### 2.6.3. Statistical Analysis: Synchrony of Feeding and Ruminating Behaviour

The present study used a novel methodology developed and described by Crump et al. [22] in its observations of behavioural synchrony. Synchrony analysis was undertaken on MooMonitor+ data for ruminating, resting and feeding behaviours. Lying behaviour could not be directly interpreted from the collars’ data output, so resting was used as a proxy. Ruminating was included in the analysis on the basis that it usually takes place when the rumen has been sufficiently filled, and disruptions to rumination may imply reduced welfare [31]. Synchrony data were prepared and analysed according to a combination of methodologies devised by Crump et al. [22] and Ruckstuhl [32]. 

In the present study, synchrony was assessed using each 15-min time interval recorded by the MooMonitor+ collars. Behaviours that occurred over 50% or more of the interval were recorded as ‘occurring’ for that animal during that interval, and any behaviour that occurred <50% of the time interval was recorded as ‘not occurring’ for that animal during this time interval. 

Once the data were transformed into a binary format, they were analysed according to the group mean methodology proposed by Ruckstuhl [32] (called ‘Ruckstuhl’s index’), which determines the extent to which an individual is synchronised to the rest of the group, in this case, the group of cows and heifers housed within each paddock (also outlined and evaluated in further detail by Asher and Collins [33]). In the present study, the behaviours performed by the majority of the group in each 15-min observation period (>50%) were calculated, and each animal was assigned a binary ‘synchrony index’ determined by whether they were or were not performing the same behaviour as the majority of the group. Individual indices were then calculated for each animal in the group as the mean of these binary values to determine the proportion of time each individual was behaving in synchrony with the rest of the group. 

To assess treatment differences in behavioural synchrony, linear mixed models were fitted to these indices separately for each behaviour. These had the same structure as for MooMonitor+ and RumiWatch behaviour proportions. The estimated variance component of the Group random effect was zero, resulting in model convergence warnings, so the Group effect was removed from the final model. Pairwise comparisons for significant treatment effects are presented in Supplementary Tables S12 and S13.

## 3. Results

### 3.1. Behavioural Observations

**Figure 2:**
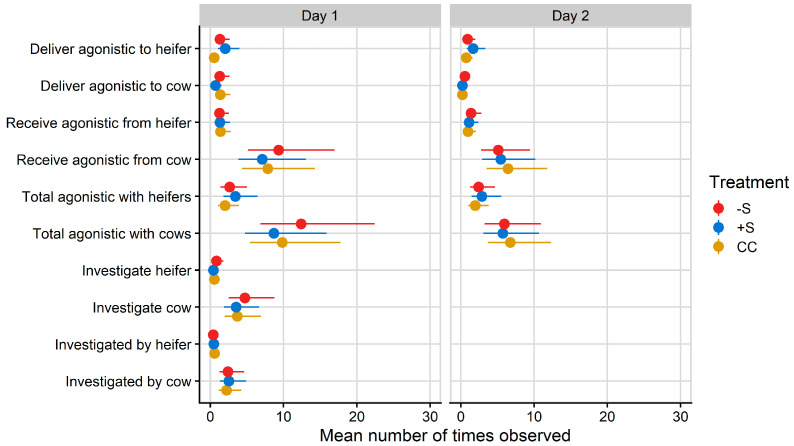
Plot of mean values/hour for social behaviours observed during the hour after mixing on Day 1 and the hour after fresh pasture allocation on Day 2 of integration testing. Total agonistic behaviour encompasses agonistic behaviours received and delivered, as well as mutual aggression (head-to-head fights). Investigative behaviour could not be included for analysis on Day 2 due to its low observance rate. CC = heifers reared commercially in sheds without adult contact; +S = heifers reared at pasture with adult contact; and −S = heifers reared at pasture without adult contact. Symbols denote the treatment mean, while spread denotes values captured within the 95% confidence interval.

Mean counts of total agonistic interactions between heifers and cows after mixing on Day 1 ranged from 8.67 for +S heifers to 12.39 for −S heifers (Figure 2).

A treatment effect was found for directing agonistic behaviours towards other heifers on Day 1 (F = 5.02, *p* = 0.007) but not on Day 2. CC heifers delivered a mean count of 0.52 agonistic behaviours per hour to other heifers on Day 1, significantly less than −S (mean count 1.30/h) and +S (mean count 2.02/h) heifers. No other differences in social behaviour were detected. 

### 3.2. Technology Data

**Figure 3:**
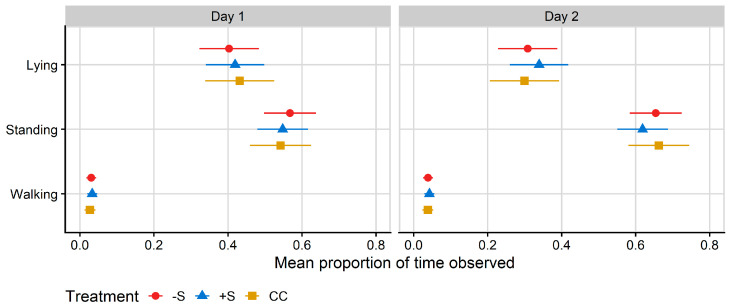
Plot of the mean proportion of time spent in each behaviour recorded by RumiWatch pedometers, per treatment. Data correspond to 24 h after first mixing with a herd of mature cows (Day 1) and 12 h after the mixed group was moved to a fresh allocation of pasture (Day 2). CC = heifers reared commercially in sheds without adult contact; +S = heifers reared at pasture with adult contact; and −S = heifers reared at pasture without adult contact. Symbols denote the treatment mean, while spread denotes values captured within the 95% confidence interval.

No differences between treatments were found for lying, standing or walking behaviours as measured by the RumiWatch pedometers. On Day 1, heifers fitted with pedometers spent 41% (means of) of their time lying, 56% of their time standing and 3% of their time walking. On Day 2, the (Figure 3) heifers spent 31% (means of) of their time lying, 65% of their time standing and 4% of their time walking. There was no treatment effect on latency to lie on either day; the mean latency to lie was 197 min on Day 1 (±33 min) and 223 min on Day 2 (±24 min). 

**Figure 4:**
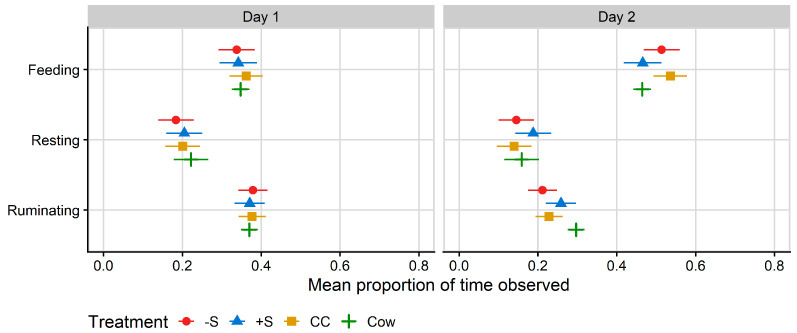
Plot of the mean proportion of time spent in each behaviour recorded by MooMonitor^+^ collars, per treatment. Data correspond to the 24 h after first mixing with a herd of mature cows (Day 1) and the 12 h after the mixed group of cows and heifers was moved to a fresh allocation of pasture (Day 2). CC = heifers reared commercially in sheds without adult contact; +S = heifers reared at pasture with adult contact; and −S = heifers reared at pasture without adult contact. Cow = resident cows taken from the larger milking herd, housed with heifers at a ratio of two cows: one heifer. Symbols denote the treatment mean, while spread denotes values captured within the 95% confidence interval.

Ruminating and feeding behaviour on Day 2 were affected by treatment (*p* < 0.001 for ruminating and *p* = 0.007 for feeding), but not on Day 1 (Table 4). Time spent feeding on Day 2 was almost identical for +S heifers, who spent 46.6% of Day 2 feeding, and cows, who spent 46.4% of Day 2 feeding. CC heifers spent a mean of 53.6% of Day 2 feeding, which was more time feeding than both cows and +S heifers (Figure 4). −S heifers spent a mean of 51.4% of Day 2 feeding, which was more than cows but not +S heifers. 

By contrast, cows spent a mean of 29.7% of Day 2 ruminating; significantly more than CC (mean 22.8% of time spent ruminating) and −S (mean 21.2% of time spent ruminating) heifers. +S heifers spent a mean of 25.8% of their time ruminating, but there were no differences in rumination between the heifer treatment groups (Figure 4). 

### 3.3. Synchrony

**Figure 5:**
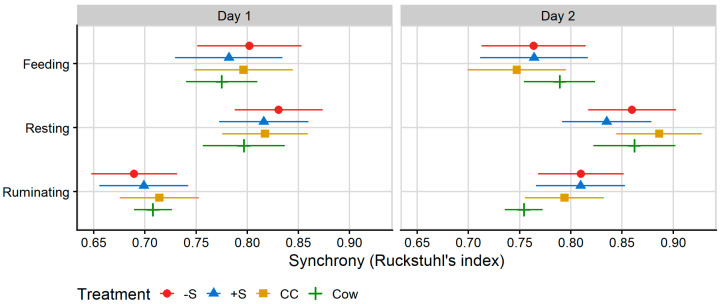
Plot of the mean proportion of time spent in synchrony with other members of the testing group for behaviours collected by MooMonitor^+^ collars across 24 h on Day 1 and 12 h on Day 2 of integration testing. Synchrony was calculated according to a binary (performing/not performing a behaviour) for each 15-min time interval collected by MooMonitor^+^ collars according to Ruckstuhl’s index ([32], i.e., a synchrony index calculated from the proportion of total time intervals spent performing the same behaviour as the group mean behaviour over the period of data collection). Data correspond to 24 h after first mixing with a herd of mature cows (Day 1) and 12 h after the mixed group was moved to a fresh allocation of pasture (Day 2). CC = heifers reared commercially in sheds without adult contact; +S = heifers reared at pasture with adult contact; and −S = heifers reared at pasture without adult contact. Cow = resident cows taken from the larger milking herd, housed with heifers at a ratio of two cows: one heifer. Symbols denote the treatment mean, while spread denotes values captured within the 95% confidence interval.

Treatment differences in behavioural synchrony were identified on Day 2 for rumination (*p* = 0.010). On Day 2, Cow rumination was significantly less synchronised with the group of cows and heifers than that of −S and +S heifers. Synchrony values are presented in Figure 5, below.

## 4. Discussion

This study aimed to examine the effects of early-life social and physical enrichment on the long-term social competency of heifers in a pasture-based dairy system specifically and the long-term behavioural development of dairy heifers more generally. Our results indicate that a combination of social and environmental enrichment during early life, provided through non-maternal adult contact and access to pasture, may influence dairy heifer integration into a herd of unfamiliar, more dominant animals, specifically dominance within the heifer group and time spent feeding and ruminating after mixing. Our results are consistent with Wagner et al. [34], who suggest that contact with mature cows (both the dam and other unrelated cows) in early life influences the behaviour of heifers during integration into a group of mature cows housed in barns. Such behavioural differences have the potential to improve heifer experiences at regrouping with older animals, usually undertaken around the commencement of first lactation, by reducing received aggression and supporting the ability to maintain feed intake. The differences observed in the present study were small, however, and thus their productivity and welfare implications should be explored in more detail moving forward.

### 4.1. Social Interactions after Regrouping

Heifers in the present study received over five times as many agonistic behaviours from cows as from other heifers. Soonberg et al. [35] similarly found heifers to receive approximately three times as much aggression per hour as cows in the same housing group. The level of agonistic behaviour observed in this pasture-based study was comparable to that observed in indoor systems. The average number of total agonistic interactions (delivered, received and mutual) between heifers and cows in the first hour after mixing in this pasture-based study was 10.3 (ranging from 8.67 for +S heifers to 12.39 for −S heifers), compared to a range of 7.2 to 9.4 per heifer reported in housed systems [17,21,36].

Introducing new members to an established herd requires the reorganisation of social relationships, inevitably involving agonistic interactions and associated social stress (review: [1]). The repertoire of agonistic interactions in cattle—including threats, displacements from desirable resources such as feed, headbutts and head-to-head fighting bouts—is often observed when unfamiliar cattle are mixed [21]. Research from housed systems indicates that the effects of aggression are more pronounced for heifers than multiparous cows, as heifers occupy lower social ranks in mixed-age herds and usually receive higher rates of aggression than cows following mixing around parturition (e.g., [34,35,37,38]; reviews: [1,16]). The effects of aggression on heifers include increased difficulty accessing desirable feed and peaceful areas to lie and rest [38,39]. Changes to social and locomotion behaviour catalysed by the introduction of new members to a group of cattle usually stabilise after 3–15 days in housed dairy systems, and newly formed social groups rarely fully stabilise within 36 h after mixing ([18,21]; review: [16,40]). The results of the present research and of others suggest that heifers can learn quickly and behaviourally adapt to new social structures, despite the social stress presented by mixing with mature cows for the first time (review: [16]). The increased space allowance and ability to access feed associated with pasture-based dairy systems compared to indoor systems may ease this social transition for animals managed outdoors.

While affiliative behaviours such as allogrooming are crucial to herd structure and directly demonstrate cohesion, they occur infrequently, which makes them difficult to use as a measure of integration in an experimental setting (review: [36]). This held true in the present study, where affiliative behaviours were rarely observed on either day of testing.

### 4.2. Effects of Early-Life Experience on Social Behaviour

CC heifers directed less agonistic behaviour towards other heifers in the 1-h post-mixing (Day 1) compared to −S or +S heifers. Within pairs of cattle, agonistic behaviours such as displacement are delivered by the dominant towards the subordinate animal [39]. The higher amount of aggression directed towards other heifers by +S and −S heifers than by CC heifers suggests heifers reared in more complex outdoor environments may become more socially competent than those reared in less complex indoor environments. Similar findings were reported by Broom and Leaver [19], who calculated the rank orders of calves reared in spatial isolation or in groups at 8 and 20 months. They found that almost all group-reared calves occupied higher dominance rankings and that these calves appeared to be more sociable, preferring to spend their time with other calves rather than alone. Le Neindre and Sourd [41] also found that Saler heifers reared by their dams displayed more agonistic behaviour than artificially reared calves, concluding that these animals were more dominant. Confirming this finding relative to the present study would require exploration of the dominance structures of the heifer herd.

This effect may also be linked to differences in space allowance during early life between the paddock-housed +S and −S heifers and the shed-housed CC heifers. Play behaviour increases with increased space allowance in the rearing environment (e.g., [42]). Higher rates of play in early life are believed to influence long-term behavioural development through locomotor, emotional or social training [42,43,44,45]. Play behaviour was not measured for all calves during early life in earlier stages of the present study, however, and thus this can only be hypothesised; the effects of early-life play on long-term social competence should be explored in future research.

We hypothesised that social facilitation and modelling during the early-life developmental period would lead +S heifers to recognise that they would not be competitive against older, larger, more dominant cows and to behave accordingly at mixing. There was no statistical indication that early-life experience shaped interactions with mature cows. This contrasts with the results of previous studies. Broom and Leaver [19], for instance, observed that low-ranked calves reared in isolation tended to initiate competitive interactions with higher-ranked group-reared peers on the day of mixing more often than calves reared socially. Wagner et al. [34] report that dam-reared calves show more submissive postures than artificially-reared calves when mixed with mature cows. A larger study with more animals and greater replication may have elucidated an effect, and future research should seek to explore this more specifically.

### 4.3. Effects of Early-Life Experience on Feeding and Rumination Behaviour 

−S and CC heifers spent less time ruminating and more time feeding than the cows on Day 2. Differences in rumination behaviour may simply reflect differences in physical size (e.g., a larger mouth size results in a larger bite size) and nutritional needs. Dong et al. [46] observed higher rumination time directly linked to higher forage intake when forage was provided at different concentrations in a TMR. First lactation heifers have a lower pasture intake than multiparous cows when housed in small groups and grazed competitively (i.e., strip-grazing [47]). In a tie-stall system, larger, older cattle consume TMR at a faster rate than younger, smaller animals and perform longer, more efficient bouts of rumination [48]. Additionally, there is evidence that social stress and competition for feed and lying spaces, as in a situation of competitive grazing, can reduce rumination time [31].

The aforementioned physiological differences between cows and heifers do not explain why the time +S heifers spent feeding and ruminating was comparable to that of cows, whereas the time spent in these behaviours by –S and CC was not. Differences in feeding behaviour between heifer treatment groups may be driven by three main factors: (1) early-life social experience enhancing heifer social skills, particularly in a competitive environment and/or (2) shaping lifelong grazing efficiency; or (3) the effect of early-life complexity on adaptability to changes in the environment. These are discussed below. 

Firstly, +S heifers may have been more socially prepared for competitive grazing with older, more dominant cattle than heifers that had not previously interacted with mature cows. Research from housed systems shows that in the day after being integrated into the milking herd, heifers have reduced access to feed and lying areas compared to mature cows ([21,38], review: [1]). Few studies have explored the acute effects of regrouping on feed intake in pasture-based systems (review: [40]). Subordinate cows continually monitor their spatial relationships relevant to those more dominant and, when grazing competitively, will reduce their bite rate, stop feeding, or move away as the proximity between them and cows that are more dominant reduces [49]. Thus, in pasture management systems that involve competitive grazing, the youngest and typically most subordinate cows in the herd may be restricted in the quantity or quality of pasture consumed and require more time to feed (e.g., [50]). In the present study, the more socially experienced +S heifers may have regulated their interactions with cows while grazing more readily than the −S and CC heifers. Consequently, the latter heifers may have experienced prolonged effects of regrouping with dominant mature cows, including continued disturbances at feeding, or may have avoided desirable grazing locations until the cows had moved on. Direct observations of heifer and cow interactions are required to confirm this; however, other research shows that heifers that experience more aggressive behaviour spend higher proportions of time feeding in the month after mixing [21].

Secondly, social modelling is known to support the development of grazing behaviour in juveniles reared in natural settings (review: [45]). Free-ranging herd-housed calves have been recorded grazing and ruminating from the age of 3 weeks, a behaviour believed to be socially facilitated by older, more experienced conspecifics (review: [45]). Arrazola et al. [51] suggest both early-life social housing and the presence of an experienced grazing companion affect the efficiency of grazing behaviour in 4-month-old heifers observed over 3 weeks. In the present study, both +S and −S heifers were reared on pasture, but only the +S heifers had grazing behaviour modelled by older animals during the early-life developmental period. While the +S heifers in the present study had similar grazing behaviour to cows when mixed into multiparous groups nearly 2 years later, the −S did not. Future research should aim to actively quantify these differences, given the importance of grazing competitiveness for dairy animal productivity in pasture-based systems.

Thirdly, a socially enriched early-life environment may have improved the behavioural plasticity of +S heifers. Broom and Leaver [19] state that the increased complexity of the early-life environment improves the individual’s behavioural flexibility and ability to respond suitably in novel situations. This is supported by research in a range of species (e.g., calves: [52], dogs: [53], humans: [54], quail: [5]). The animal welfare and productivity implications of promoting behavioural plasticity in dairy cattle may include an improved ability to adapt to the milking herd and novelty of the milking environment and equipment, including robotic milking systems and pasture-based systems, which may also improve adaptability to rotational grazing.

### 4.4. Synchrony

Treatment differences in synchrony were only observed for rumination behaviour. Cows were less synchronised with the group on Day 2 than −S or +S heifers, but not CC heifers. Feeding and lying are highly motivated and necessary maintenance behaviours, widely regarded as the most suitable measures for the analysis of herd synchrony [20,22,23,24,55]. Synchrony of rumination has not previously been calculated, and given its less social nature, its relevance in the broader scope of cow social behaviour is unclear.

Individual deviations from the collective behaviour of the group indicate whether individuals are willing and able to respond to the behaviour of other animals by adopting the same behaviour themselves [24]. A reduction in synchronous lying and feeding behaviour may indicate reduced welfare, due, for instance, to increased disturbance and competition for space and desirable areas in more intensive systems [55,56]. Stoye et al. [57] suggest that a synchrony threshold of 70% is appropriate for cattle housed in pasture. In the present study, animals of all treatments were in synchrony with the rest of the animals in their paddocks > 70% of the time on both days and for all behaviours, excluding rumination of +S and −S heifers on Day 1, suggesting that most experimental animals quickly acclimatised to their new social groups.

### 4.5. Implications for Pasture-Based Systems

The longitudinal results presented in this study are particularly relevant to the dairy industry, in which most replacement heifers will not come into social contact with older animals until their first calving period. Observed treatment differences in the present study were relatively minor, and their sustained benefits for the production and welfare of heifers integrating into the milking herd are unclear. Future research should aim to elucidate the tangible welfare benefits that may be afforded by the treatment differences identified in this study.

The results of the present study suggest that primiparous heifers show rapid behavioural adaptation when mixed with multiparous cows for the first time at pasture. Intensive grazing is a major feature of pastoral dairy systems; however, greater space allowances compared to indoor housing systems and the provision of feed across the entire housing area (rather than at a point location) may mitigate some of the competition and associated stress around regrouping [40]. For example, this study observed high levels of synchrony for feeding and resting behaviour across both days of testing, generally above previously published thresholds [57]. This suggests that animals of all treatments were able to meet their maintenance needs and could rest comfortably with little competition for space. 

Additionally, early-life treatment did not affect the total time spent lying after integration in the present study; similarly, few effects of regrouping on lying time were found by Wagner et al. [34], Gonzalez et al. [38] or Boyle et al. [21], who conducted similar integration studies in indoor systems. Latency to lie in the present study was, however, shorter than that recorded by Wagner et al. [34], in which only four of 26 experimental heifers were laid down within 6 h of mixing. Compared to indoor housing systems, the pasture-based environment may provide heifers and other subordinate cows with a greater opportunity to access high-priority resources such as lying space and feed. Pasture-based systems do appear to result in fewer observed day-to-day agonistic interactions between animals, likely due to the greater provision of space per animal (review: [58]). Treatment differences in heifer behaviour may be more apparent with restricted feed and space availability. 

## 5. Conclusions

The results of the present study support our hypothesis that social modelling of appropriate behaviours can shape longitudinal behavioural patterns and social competence in heifers. Heifers reared with adult cows at pasture had a behavioural profile most similar to that of the resident mature cows with which they were mixed. Early-life social complexity may have improved the ability and speed of these heifers to adapt to grazing in a group of dominant multiparous cattle, and future research should aim to elucidate the implications of these behavioural differences for the welfare and productivity of first-calving dairy heifers.

## Figures and Tables

**Table 1:** Heifer management stages.

Age	0–2 Weeks ^1^	2–13 Weeks ^1^	13 Weeks–18 Months	18 Months	18 Months–23 Months	23 Months
+S ^2^ (*n* = 20)	Sheds	Experimental—at pasture with cows	Mixed and housed at pasture	Pregnancy Testing. Remaining: +S *n* = 16 −S *n* = 17 CC *n* = 17	Mixed and housed at pasture	Integration testing with multiparous animals
−S (*n* = 20)	Sheds	Experimental—at pasture				
CC (*n* = 20)	Sheds	Retained in sheds				

^1^ Management and treatment details for +S and −S heifers up to the age of 13 weeks are outlined in full in Field et al., 2023 [26]. ^2^ CC = heifers reared commercially in sheds without adult contact; +S = heifers reared at pasture with adult contact; and −S = heifers reared at pasture without adult contact.

**Table 2:** Group composition for each paddock during integration testing.

Time Replicate	Paddock/Group	Heifers				Cows ^3^
		+S	−S	CC	Total	
1 (EC) ^1^	1 ^2^	2	4	3	9	18
	2	2	2	3	7	14
	3	2	2	3	7	14
2 (LC)	1	4	4	3	11	22
	2	4	3	3	10	20
	3	2	2	2	6	12

This table describes the number of heifers from each treatment (+S, −S and CC) and the corresponding number of cows which together made up each group during testing. The paddocks in that each group was tested can be seen in Figure 1, wherein ‘EC1′ corresponds to Group 1 from the EC time replicate. ^1^ Testing was undertaken in 2 time-replicates according to estimated heifer calving dates, wherein EC = early calving time-replicate and LC = late calving time replicate. ^2^ Each testing time-replicate comprised 3 groups of heifers, balanced as best as possible for treatment. Treatment distribution per heifer group is outlined in the table. ^3^ Each heifer testing group was mixed with a resident group of mature cows from the research farm’s milking herd at a ratio of 2 cows per heifer. The number of cows in each resident group is outlined in this column.

**Table 3:** Ethogram of behaviours observed in heifers during two 1-h periods when mixed with mature cows ^1^.

Behaviour		Definition
Deliver	Displacement	The heifer turns towards or approaches a cow/heifer with her head down and then lunges without making contact, actively moves towards a cow/heifer, causing this individual to retreat, or physically displaces a cow/heifer using any part of their body except the front of the head.
	Butting	The heifer uses the front of her head to make vigourous contact with any area of a cow/heifer’s body—a blow with the forehead directed at another cow/heifer.
	Cohesive	The heifer licks or rubs against a cow/heifer without displaying agonistic behaviour (not reciprocated), licking another cow/heifer’s head, neck and/or shoulder areas.
	Investigate	Head or muzzle stretched towards or even touching another cow/heifer’s body or head; nosing or sniffing any part of a cow/heifer without displaying agonistic or cohesive behaviour.
Receive	Displacement	Another cow/heifer turns towards or approaches the heifer with her head down and then lunges without making contact, actively moves towards the heifer, causing this individual to retreat, or physically displaces the heifer using any part of their body except the front of the head.
	Butting	Another cow/heifer uses the front of her head to make vigourous contact with any area of the heifer’s body—a blow with the forehead directed at the heifer.
	Cohesive	Another cow/heifer licks or rubs against the heifer without displaying agonistic behaviour (not reciprocated).
	Investigate	Another cow/heifer noses or sniffs any part of the heifer without displaying agonistic or cohesive behaviour.
Mutual	Allogrooming	Mutual grooming between 2 individuals—a shared behaviour
	Head-to-head contact	Contact between two animals, where both push against the front of one another’s heads using the front of their own heads. This was considered a single mutual interaction (i.e., neither delivered nor received) until contact between front of heads was disturbed.

^1^ Ethogram developed with reference to Boyle et al. (2012 [21]).

**Table 4:** Overall test for the effect of treatment on behaviour captured by MooMonitor+ collars per observation day ^a^.

Behaviour	Day 1		Day 2	
	F (df)	*p*	F (df)	*p*
Ruminating	0.1 (3, 196)	0.958	10 (3, 196)	<0.001 *
Resting	2.3 (3, 201)	0.079 **	2 (3, 201)	0.111
Feeding	0.2 (3, 202)	0.872	4.1 (3, 202)	0.007 *

^a^ This table shows the results of overall tests for the effect of treatment, within each behaviour and observation day. Data correspond to 24 h after first mixing with a herd of mature cows (Day 1) and 12 h after the mixed group was moved to a fresh allocation of pasture (Day 2). * signifies a significant difference as determined by statistical analysis (i.e., *p* < 0.05); ** signifies a statistical tendency wherein 0.05 < *p* < 0.1.

## Data Availability

Data are available on reasonable request from the authors.

## Supplementary Materials

The following supporting information can be downloaded at: https://www.mdpi.com/article/10.3390/ani13132049/s1, Table S1: Table outlining management procedures and data collection over the testing period of the integration trial; Table S2: Table of means detailing mean count of observed social behaviours over one 1 h period on Day 1, and one 1hr period on Day 2; Table S3: Table detailing results of overall tests for effect of treatment on the nature of heifer interactions during testing; Table S4: Table detailing pairwise comparisons of delivering agonistic behaviours to other heifers (Day 1); Table S5: Table of means detailing mean proportion of time spent in behaviours recorded by RumiWatch pedometers; Table S6: Overall test for effect of treatment on behaviour captured by RumiWatch pedometers per observation day; Table S7: Table detailing results of pairwise comparisons for significant behaviours (RumiWatch Pedometers); Figure S1: Figure illustrating mean latency to lie; Table S8: Table of means detailing mean latency to lie; Table S9: Table detailing results of pairwise comparisons for latency to lie; Table S10: Table of Means—MooMonitor collars; Table S11: Pairwise comparisons (MooMonitor Collars); Table S12: table detailing pairwise comparisons of synchrony data; Table S13. Overall test for effect of treatment on synchrony behaviour per observation day.
