# Supplementary material for: Contact with Mature Cows and Access to Pasture during Early Life Shape Dairy Heifer Behaviour at Integration into the Milking Herd
Source: Animals (Basel). 2023 Jun 21;13(13):2049. doi: 10.3390/ani13132049 (PMC10339967; doi:10.3390/ani13132049)
Supplement: Supplementary file 1 [file animals-13-02049-s001.zip › animals-2381250-supplementary.pdf]

Supplementary Table S1: Table outlining management procedures and data collection over the testing period of the integration trial

Day	Heifers	Cows	Data Collected
-4[1]	Housed as one large replacement heifer herd	Housed in two large groups – early calvers and late calvers	None
-3	Replicate (EC or LC[2]) for weekend testing drafted from larger replacement herd. Heifers drafted individually into testing groups and sprayed to identify. Groups housed at pasture, no visual access to testing paddocks.	Housed in two large groups – early calvers and late calvers	None
-2	Groups housed at pasture with no visual access to testing paddocks.	Housed in two large groups – early calvers and late calvers	None
-1	Groups housed at pasture with no visual access to testing paddocks.	Replicate (EC or LC[2]) brought to stockyards. Allocated cows sprayed to identify, drafted into 3 groups. Each group housed in section 1 of testing paddock.	None
1	Heifers walked down laneway to testing paddock, walked through section 1 and into section 2, already populated with cows.	Cows given access to section 2 of testing paddock	Heifers: collar & pedometer, continuous 1hr continuous observation of integration behaviour immediately after mixing<br>Cows: collars, continuous
2	All animals given access to section 3 of testing paddock		Heifers: collar & pedometer, continuous; 1 hour continuous observation of integration behaviour immediately after move to fresh pasture in section 3.<br>Cows: collars continuous
+1	Heifers drafted from testing groups and returned to farm for entry into pre-calving lead feed program.	Cows drafted from testing groups and returned to larger management groups.	None

[1]Day 1 denotes the first day of testing, and is described as 'Day 1' in the body of the paper. Management in the periods leading up to testing are denoted by the (-) symbol. Management in the days following the integration trial are denoted by the (+) symbol.
[2]EC denotes early calvers – animals due to calve in the first calving peak of the seasonal calving block. LC denotes late calvers – animals due to calve in the second calving peak of the seasonal calving block.

Supplementary Table S2: Table of means detailing mean count of observed social behaviours over one 1hr period on Day 1, and one 1hr period on Day 2[1]

Day	Behaviour	Partner	Direction	Treatment	Mean Count	95% CI
1	**Agonistic**	**Heifer**	**Deliver**	-S	1.30	0.64, 2.63
				+S	2.02	1.03, 3.97
				CC	0.52	0.22, 1.23
			**Receive**	-S	1.25	0.62, 2.54
				+S	1.31	0.64, 2.67
				CC	1.38	0.68, 2.77
			**Mutual/Total**	-S	2.62	1.37, 5.00
				+S	3.40	1.80, 6.45
				CC	2.01	1.03, 3.90
		**Cow**	**Deliver**	-S	1.28	0.63, 2.60
				+S	0.70	0.31, 1.55
				CC	1.34	0.66, 2.72
			**Receive**	-S	9.32	5.12, 16.96
				+S	7.09	3.84, 13.06
				CC	7.83	4.30, 14.26
			**Mutual/Total**	-S	12.39	6.84, 22.43
				+S	8.67	4.73, 15.88
				CC	9.80	5.41, 17.74
	**Investigate**	**Heifer**	**Deliver**	-S	0.82	0.38, 1.78
				+S	0.42	0.17, 1.03
				CC	0.54	0.23, 1.26
			**Receive**	-S	0.39	0.16, 0.97
				+S	0.45	0.19, 1.10
				CC	0.58	0.25, 1.33
		**Cow**	**Deliver**	-S	4.69	2.52, 8.75
				+S	3.51	1.85, 6.66
				CC	3.68	1.96, 6.91
			**Receive**	-S	2.39	1.24, 4.60
				+S	2.52	1.30, 4.89
				CC	2.20	1.14, 4.25
2	**Agonistic**	**Heifer**	**Deliver**	-S	0.93	0.44, 1.98
				+S	1.67	0.83, 3.37
				CC	0.74	0.34, 1.63
			**Receive**	-S	1.40	0.70, 2.82
				+S	1.14	0.54, 2.40
				CC	0.98	0.47, 2.06
			**Mutual**	-S	2.44	1.27, 4.69
				+S	2.86	1.48, 5.53
				CC	1.96	1.01, 3.82
		**Cow**	**Deliver**	-S	0.55	0.24, 1.26
				+S	0.21	0.07, 0.66
				CC	0.23	0.08, 0.68
			**Receive**	-S	5.13	2.78, 9.47
				+S	5.45	2.91, 10.19
				CC	6.45	3.53, 11.80
			**Mutual**	-S	5.96	3.24, 10.94
				+S	5.73	3.07, 10.69
				CC	6.74	3.69, 12.31

[1] This table provides mean values for social behaviours observed during the hour after mixing on Day 1, and the hour after fresh pasture allocation on Day 2 of integration testing. Total agonistic behaviour encompasses agonistic behaviours received and delivered, as well as mutual aggression (head-to-head fights). Investigative behaviour could not be included for analysis on Day 2 due its low observance rate. CC= heifers reared commercially in sheds, without adult contact; +S= heifers reared at pasture with adult contact and -S= heifers reared at pasture without adult contact.

Supplementary Table S3: Table detailing results of overall tests for effect of treatment on the nature of heifer interactions during testing[1]

Day	Behaviour	Partner	Direction	*p*	F
1	Agonistic	Heifer	Deliver	**0.007***	5.02
			Receive	0.965	0.04
			Mutual	0.233	1.46
		Cow	Deliver	0.226	1.49
			Receive	0.555	0.59
			Mutual	0.343	1.07
	Investigate	Heifer	Deliver	0.329	1.11
			Receive	0.729	0.32
		Cow	Deliver	0.532	0.63
			Receive	0.913	0.09
**2**	Agonistic	Heifer	Deliver	0.101	2.30
			Receive	0.636	0.45
			Mutual	0.498	0.70
		Cow	Deliver	0.169	1.78
			Receive	0.662	0.41
			Mutual	0.812	0.21

[1]Data for each day correspond with the first hour after: Day 1: Heifers mixing with cows and, Day 2: Group allocation of fresh pasture. Observations were made by a single observer per heifer only. Significance is determined where $p<0.05$, and is denoted in bold with an asterisk. Df for all tests= 2, 925.

Supplementary Table S4: Table detailing pairwise comparisons of delivering agonistic behaviours to other heifers (Day 1)[1]

**Measure**	**(+S) / (-S)**	**CC / (-S)**	**CC / (+S)**
Ratio of means	1.6	0.40	0.26
95% CI	0.79, 3.1	0.17, 0.97	0.11, 0.60
p-value	0.203	**0.041***	**0.002***

[1]Data is provided only for pairwise tests undertaken on data sets where overall tests for effect of treatment yielded a significant result. Significance is determined where $p<0.05$, and is denoted in bold with an asterisk. CC= heifers reared commercially in sheds, without adult contact; +S= heifers reared at pasture with adult contact and -S= heifers reared at pasture without adult contact.

**Supplementary Table S5: Table of means detailing mean proportion of time spent in behaviours recorded by RumiWatch pedometers[1]**				
**Day**	**Behaviour**	**Treatment**	**Mean proportion of time**	**95% CI**
**Day 1**	**Lying**	-S	0.403	0.323, 0.483
		+S	0.419	0.340, 0.498
		CC	0.432	0.338, 0.525
	**Standing**	-S	0.568	0.497, 0.638
		+S	0.548	0.479, 0.616
		CC	0.542	0.460, 0.625
	**Walking**	-S	0.031	0.017, 0.044
		+S	0.033	0.020, 0.046
		CC	0.027	0.013, 0.042
**Day 2**	**Lying**	-S	0.308	0.228, 0.389
		+S	0.339	0.260, 0.418
		CC	0.300	0.206, 0.393
	**Standing**	-S	0.654	0.584, 0.724
		+S	0.619	0.550, 0.687
		CC	0.663	0.580, 0.745
	**Walking**	-S	0.039	0.025, 0.052
		+S	0.042	0.029, 0.056
		CC	0.038	0.024, 0.053

[1] This table provides mean values for data collected by RumiWatch pedometers fitted to 36 experimental heifers for 24h after mixing on Day 1, and 12h after fresh pasture allocation on Day 2 of integration testing. CC= heifers reared commercially in sheds, without adult contact; +S= heifers reared at pasture with adult contact and -S= heifers reared at pasture without adult contact.

Supplementary Table S6: Overall test for effect of treatment on behaviour captured by RumiWatch pedometers per observation day

**Behaviour**	**Day 1**		**Day 2**	
	**F (df)**	***p***	**F (df)**	***p***
Lying	0.2 (2, 36)	0.809	0.5 (2, 36)	0.632
Standing	0.3 (2, 36)	0.759	1.0 (2, 36)	0.477
Walking	0.4 (2, 36)	0.714	0.3 (2, 36)	0.786

**Supplementary Table S7: Table detailing results of pairwise comparisons for significant behaviours (RumiWatch Pedometers)[1]**					
**Day**	**Behaviour**	**Pairwise**	**Difference of mean proportion[2]**	**95% CI**	***p***
**Day 1**	**Lying**	(+S) - (-S)	0.016	−0.102, 0.134	0.688
		CC - (-S)	0.029	−0.093, 0.150	0.552
		CC - (+S)	0.013	−0.108, 0.133	0.789
	**Standing**	(+S) - (-S)	−0.020	−0.118, 0.078	0.566
		CC - (-S)	−0.026	−0.129, 0.077	0.539
		CC - (+S)	−0.006	−0.107, 0.096	0.887
	**Walking**	(+S) - (-S)	0.003	−0.017, 0.022	0.696
		CC - (-S)	−0.003	−0.023, 0.016	0.652
		CC - (+S)	−0.006	−0.025, 0.013	0.436
**Day 2**	**Lying**	(+S) - (-S)	0.031	−0.087, 0.148	0.461
		CC - (-S)	−0.009	−0.130, 0.113	0.855
		CC - (+S)	−0.039	−0.160, 0.081	0.421
	**Standing**	(+S) - (-S)	−0.036	−0.134, 0.063	0.333
		CC - (-S)	0.009	−0.094, 0.112	0.833
		CC - (+S)	0.044	−0.057, 0.146	0.305
	**Walking**	(+S) - (-S)	0.004	−0.016, 0.023	0.575
		CC - (-S)	0.000	−0.020, 0.019	0.969
		CC - (+S)	−0.004	−0.023, 0.015	0.585

[1]Data is provided only for pairwise tests undertaken on data sets where overall tests for effect of treatment yielded a significant result. Significance is determined where $p<0.05$, and is denoted in bold with an asterisk. CC= heifers reared commercially in sheds, without adult contact; +S= heifers reared at pasture with adult contact and -S= heifers reared at pasture without adult contact. [2]Proportion of time.

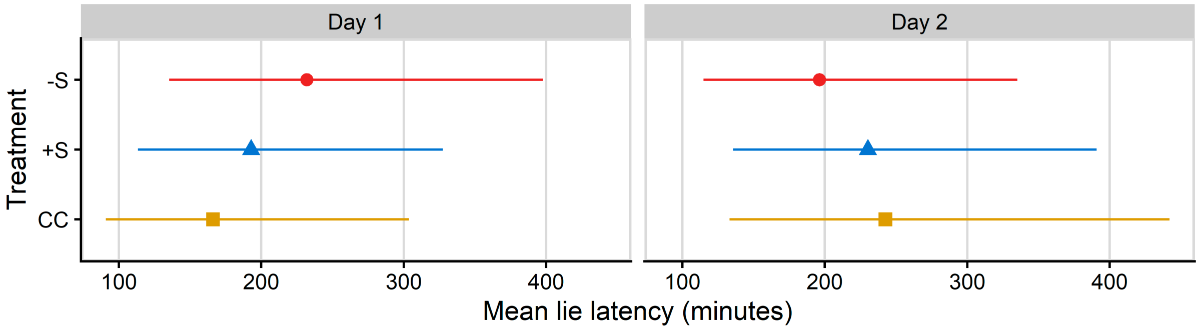

Supplementary Figure S1: Figure illustrating mean latency to lie[1]

[1] CC= heifers reared commercially in sheds, without adult contact; +S= heifers reared at pasture with adult contact and -S= heifers reared at pasture without adult contact.

Supplementary Table S8: Table of means detailing mean latency to lie[1]

Day	Treatment	Mean proportion of time	95% CI
**1**	-S	232	135, 398
	+S	193	113, 327
	CC	166	91, 304
**2**	-S	196	115, 335
	+S	230	136, 391
	CC	243	133, 442

[1] Data in this table was collected by RumiWatch pedometers fitted to 36 experimental heifers for 24h after mixing on Day 1, and 12h after fresh pasture allocation on Day 2 of integration testing. CC= heifers reared commercially in sheds, without adult contact; +S= heifers reared at pasture with adult contact and -S= heifers reared at pasture without adult contact.

Supplementary Table S9: Table detailing results of pairwise comparisons for latency to lie[1]

Day	Pairwise	Ratio of Means[2]	95% CI	*p*
**1**	(+S) / (-S)	0.83	0.56, 1.24	0.358
	CC / (-S)	0.72	0.45, 1.14	0.154
	CC / (+S)	0.86	0.53, 1.40	0.542
**2**	(+S) / (-S)	1.17	0.79, 1.74	0.423
	CC / (-S)	1.24	0.78, 1.96	0.357
	CC / (+S)	1.05	0.65, 1.71	0.828

[1]Data is provided for pairwise tests undertaken on all latency to lie data. Data in this table was collected by RumiWatch pedometers fitted to 36 experimental heifers for 24h after mixing on Day 1, and 12h after fresh pasture allocation on Day 2 of integration testing. Significance is determined where $p<0.05$, and is denoted in bold with an asterisk. CC= heifers reared commercially in sheds, without adult contact; +S= heifers reared at pasture with adult contact and -S= heifers reared at pasture without adult contact.

[2]Proportion of time

Supplementary Table S10: Table of Means – MooMonitor collars[1]				
**Day**	**Behaviour**	**Treatment**	**Mean proportion of time**	**95% CI**
**Day 1**	**Rumination**	-S	0.379	0.342, 0.416
		+S	0.371	0.332, 0.409
		CC	0.377	0.342, 0.411
		Cow	0.370	0.349, 0.391
	**Resting**	-S	0.184	0.139, 0.229
		+S	0.205	0.159, 0.250
		CC	0.201	0.156, 0.245
		Cow	0.222	0.178, 0.266
	**Feeding**	-S	0.338	0.292, 0.384
		+S	0.342	0.294, 0.389
		CC	0.362	0.319, 0.404
		Cow	0.348	0.325, 0.370
**Day 2**	**Rumination**	-S	0.212	0.175, 0.249
		+S	0.258	0.220, 0.296
		CC	0.228	0.194, 0.263
		Cow	0.297	0.276, 0.318
	**Resting**	-S	0.145	0.100, 0.190
		+S	0.188	0.142, 0.233
		CC	0.140	0.096, 0.184
		Cow	0.159	0.115, 0.203
	**Feeding**	-S	0.514	0.468, 0.560
		+S	0.466	0.418, 0.513
		CC	0.536	0.494, 0.578
		Cow	0.464	0.442, 0.587

[1] This table provides mean values for data collected by MooMonitor+ collars fitted to all experimental animals, for 24h after mixing on Day 1, and 12h after fresh pasture allocation on Day 2 of integration testing. CC= heifers reared commercially in sheds, without adult contact; +S= heifers reared at pasture with adult contact and -S= heifers reared at pasture without adult contact, Cow = resident cows taken from the larger milking herd, housed with heifers at a ratio of 2 cows:1 heifer.

Supplementary Table S11: Pairwise comparisons (MooMonitor Collars)[1]					
**Day**	**Behaviour**	**Pairwise**	**Difference of means[2]**	**95% CI**	***p***
**Day 1**	**Rumination**	(+S) - (-S)	−0.008	−0.059, 0.043	0.754
		CC - (-S)	−0.002	−0.050, 0.046	0.936
		CC - (+S)	0.006	−0.043, 0.055	0.805
		Cow - (-S)	−0.009	−0.047, 0.029	0.649
		Cow - (+S)	−0.001	−0.040, 0.039	0.971
		Cow - CC	−0.007	−0.043, 0.029	0.704
	**Resting**	(+S) - (-S)	0.021	−0.022, 0.064	0.339
		CC - (-S)	0.017	−0.024, 0.057	0.413
		CC - (+S)	−0.004	−0.045, 0.037	0.848
		Cow - (-S)	0.038	0.006, 0.070	**0.020****
		Cow – (+S)	0.017	−0.016, 0.051	0.307
		Cow – CC	0.021	−0.009, 0.051	0.164
	**Feeding**	(+S) – (-S)	0.004	0.061, 0.069	0.902
		CC – (-S)	0.024	−0.037, 0.085	0.439
		CC – (+S)	0.020	−0.042, 0.082	0.529
		Cow – (-S)	0.010	−0.039, 0.058	0.688
		Cow – (+S)	0.006	−0.044, 0.056	0.818
		Cow – CC	-0.014	−0.060, 0.031	0.541
**Day 2**	**Rumination**	(+S) – (-S)	0.047	−0.004, 0.097	*0.072**
		CC - (-S)	0.017	−0.031, 0.064	0.496
		CC - (+S)	−0.030	−0.079, 0.019	0.227
		Cow - (-S)	0.085	0.047, 0.123	**<0.001****
		Cow – (+S)	0.038	−0.001, 0.078	*0.056**
		Cow – CC	0.069	0.033, 0.10	**<0.001****
	**Resting**	(+S) – (-S)	0.043	0.000, 0.086	**0.050****
		CC – (-S)	−0.005	−0.046, 0.035	0.797
		CC – (+S)	−0.048	−0.089, −0.007	**0.023****
		Cow – (-S)	0.014	−0.018, 0.046	0.402
		Cow – (+S)	−0.029	−0.062, 0.004	*0.086**
		Cow – CC	0.019	−0.011, 0.049	0.215
	**Feeding**	(+S) – (-S)	0.048	−0.113, 0.017	0.144
		CC – (-S)	0.022	−0.039, 0.083	0.473
		CC – (+S)	0.070	0.008, 0.133	**0.027****
		Cow – (-S)	−0.049	−0.098, −0.001	**0.046****
		Cow – (+S)	−0.001	−0.051, 0.049	0.961
		Cow – CC	−0.072	−0.117, −0.026	**0.002****

[1]Data is provided for pairwise tests undertaken on all data collected by MooMonitor+ collars fitted to all experimental animals, for 24h after mixing on Day 1, and 12h after fresh pasture allocation on Day 2 of integration testing. Significance is determined where p<0.05, and is denoted in bold with an asterisk. CC= heifers reared commercially in sheds, without adult contact; +S= heifers reared at pasture with adult contact and -S= heifers reared at pasture without adult contact, Cow = resident cows taken from the larger milking herd, housed with heifers at a ratio of 2 cows:1 heifer.

[2]Proportion of time

**Supplementary Table S12: table detailing pairwise comparisons of synchrony data**[1]				
**Day**	**Pairwise**	**Ratio of Means**	**95% CI**	***p***
**2**	(+S) - (-S)	0.000	−0.061, 0.060	0.990
	CC - (-S)	−0.016	−0.073, 0.041	0.579
	CC - (+S)	−0.016	−0.074, 0.042	0.596
	Cow - (-S)	−0.056	−0.101, −0.011	**0.016****
	Cow – (+S)	−0.055	−0.102, −0.009	**0.020****
	Cow – CC	−0.040	−0.082, 0.002	*0.064**

[1] Data is provided only for pairwise tests undertaken on data sets where overall tests for effect of treatment yielded a significant result. Synchrony was calculated according to a binary (performing/not performing a behaviour) for each 15min time interval collected by MooMonitor+ collars according to Ruckstuhl's index (i.e. a synchrony index calculated from the proportion of total time intervals spent performing the same behaviour as the group mean behaviour over the period of data collection). Data correspond to the 24h after first mixing with a herd of mature cows (Day 1) and 12h after the mixed group was moved to a fresh allocation of pasture (Day 2). CC= heifers reared commercially in sheds, without adult contact; +S= heifers reared at pasture with adult contact and -S= heifers reared at pasture without adult contact; Cow = resident cows taken from the larger milking herd, housed with heifers at a ratio of 2 cows:1 heifer.

Supplementary Table S13. Overall test for effect of treatment on synchrony behaviour per observation day[a.]

**Behaviour**	**Day 1**		**Day 2**	
	**F (df)**	***p***	**F (df)**	***p***
Ruminating	0.3 (3, 202)	0.819	3.9 (3, 202)	**0.010***
Resting	1.9 (3, 201)	0.130	1.9 (3, 201)	0.128
Feeding	0.6 (3, 201)	0.629	1.4 (3, 201)	0.248

[a] Data correspond to the 24h after first mixing with a herd of mature cows (Day 1) and 12h after the mixed group was moved to a fresh allocation of pasture (Day 2). *signifies a significant difference as determined by statistical analysis (i.e. $p<0.05$).
